# Fisetin Attenuates Amyloid‐Beta‐Induced Neurotoxicity in Human Neuroblastoma SH‐SY5Y Cells: Integrating In Silico Target Prediction and In Vitro Validation

**DOI:** 10.1002/jbt.71076

**Published:** 2026-08-17

**Authors:** Charu Jaiswal, Ishika Singh, Abhishek Kumar Singh

**Affiliations:** ^1^ Department of Biotherapeutics Research Manipal Academy of Higher Education Manipal India

**Keywords:** amyloid‐beta, fisetin, molecular docking, molecular dynamics simulation, neuroprotection, neurotoxicity

## Abstract

The accumulation of amyloid beta (Aβ) and tau tangles in the brain leads to Alzheimer's disease (AD). Fisetin, a natural flavonoid, is an antioxidant molecule, and its neuroprotective effects are not clearly understood. Therefore, attempts have been made to evaluate the neuroprotective effects of fisetin using in silico methods and an Aβ1‐42‐induced neurotoxicity model in human neuroblastoma SH‐SY5Y cells. In silico studies demonstrated that fisetin binds strongly and with high stability to different proteins, such as ULK1 (autophagy marker), p21 (senescence/cell cycle marker), and synaptophysin (synaptic marker), which are responsible for maintaining brain health and are implicated in AD. Moreover, Aβ1‐42 was also found to bind to these protein targets, indicating that Aβ1‐42 and fisetin both target common binding sites. In vitro studies on SH‐SY5Y cells further confirmed that fisetin promotes cell survival under the toxic effects of Aβ1‐42. It reduced oxidative stress and restored the activities of ion channels, which were impaired by Aβ1‐42 treatment. Fisetin increased antioxidant defense and restored the activity of molecules that control brain signals. Overall, fisetin acts on multiple targets to protect neurons by reducing oxidative damage, supporting ion channel activity, and inducing the autophagy process.

## Graphical abstract

The in‐silico data showed that fisetin binds stably with the autophagy receptors and synaptic density regulators. Further, in‐vitro data confirmed the restoration of Aβ1‐42 disrupted autophagy by fisetin. Fisetin restored the oxidative stress and ion channels, along with a reduction of AChE activity.

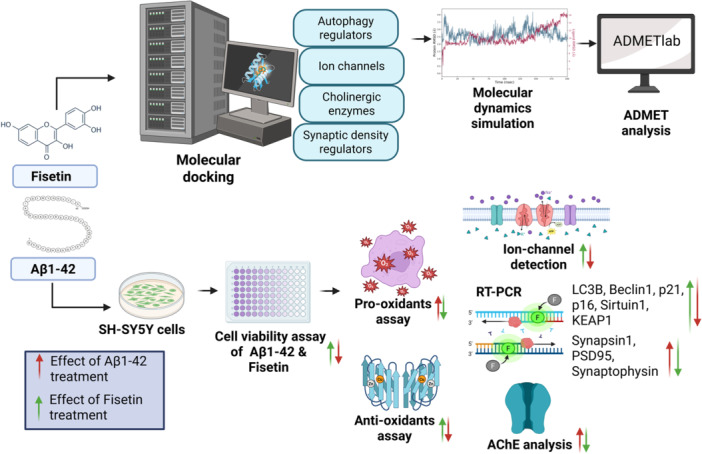

## 1 Introduction

Alzheimer's disease (AD) is caused by the buildup of amyloid‐beta (Aβ) peptides from amyloid precursor protein (APP) into senile plaques [1, 2] and the formation of neurofibrillary tangles (NFTs) due to the aggregation of hyperphosphorylated tau protein [3]. Recent findings have suggested that Aβ pathology generally precedes tau changes. Aβ triggers tau phosphorylation and enhances its aggregation, which is a significant step in AD progression. These molecular changes disrupt synaptic function and lead to neuronal loss and brain atrophy, particularly in the hippocampus and cortex, often through mechanisms involving excitotoxicity, synaptic dysfunction, and oxidative stress [4, 5]. Aβ often disrupts calcium homeostasis and promotes the generation of free radicals that lead to damage to mitochondria and macromolecules in neurons [6].

Autophagy is an essential process that helps in clearing damaged proteins and organelles, decreases with age, and leads to protein aggregation in AD [7, 8]. The mTOR pathway, particularly mTORC1, regulates autophagy and cellular homeostasis, and its modulation influences neurodegeneration [9, 10]. Pharmacological compounds such as fisetin, an autophagy inducer, can modulate the activity of the PI3K/AKT/mTOR signaling pathway to induce autophagy [11].

Fisetin, a bioactive flavonoid, has therapeutic potential in various inflammatory and neurodegenerative conditions and is known to improve cognitive deficits. At the molecular level, fisetin reduces neuroinflammation through the downregulation of proinflammatory markers, such as pNF‐κB, IL‐1R1, TNF‐α, and iNOS, in microglia. It promotes mitophagy, which leads to a reduction in reactive oxygen species (ROS) generation. Moreover, it also inhibits NLRP3 inflammasome activation in cerebral microvascular endothelial cells (CMECs), reducing the release of IL‐1β into the CNS. These findings demonstrate that fisetin may protect against cognitive impairment by modulating mitochondrial activity and suppressing neuroinflammatory signaling pathways [12].

Moreover, fisetin significantly reduced neurological damage, as shown by improved neurobehavioral scores, decreased brain edema, and reduced neuronal loss, as shown by Nissl, H&E, and TUNEL staining experiments, as well as enhanced cognitive performance in the Morris water maze test. Fisetin often activates the PI3K/AKT/NRF2 signaling pathway at the molecular level, thereby reducing oxidative stress [13]. Fisetin promotes the autophagic degradation of phosphorylated tau by activating the transcription factors TFEB and Nrf2. This activation enhances the expression of autophagy‐lysosome pathway genes, which facilitates the clearance of toxic tau aggregates associated with AD. This process is closely linked to fisetin‐mediated inhibition of mTORC1, which further leads to autophagy activation, reducing phosphorylated tau levels in neuronal cells [14].

The exposure to Aβ has been shown to significantly compromise neuronal viability, primarily through enhanced oxidative stress, mitochondrial dysfunction, and the activation of apoptotic pathways. Treatment with fisetin attenuated these effects, suggesting the strong cytoprotective ability of fisetin. Fisetin has been shown to restore redox balance by reducing intracellular ROS levels and improving mitochondrial integrity. In addition, it modulated key apoptotic markers, indicating a shift from pro‐apoptotic to survival signaling. These findings underscore the therapeutic importance of fisetin in mitigating Aβ‐associated neurotoxicity and suggest its potential utility in slowing the progression of neurodegenerative conditions, such as AD [15].

While studies have investigated shorter Aβ peptides such as Aβ25‐35 [16] and their neurotoxicity, studies on the Aβ1‐42 peptide and its effects are limited. The inhibition of Aβ fibril formation by fisetin has been studied [17], but studies on its mechanism are limited. Therefore, we performed in silico studies to examine the interactions of fisetin with autophagy markers, synaptic markers, cholinergic enzymes, and ion channels. Furthermore, we investigated the effects of fisetin on Aβ1‐42‐intoxicated SH‐SY5Y cells through in vitro experiments and assessed oxidative stress, ion channel function, and gene expression of key autophagy and synaptic markers to establish a connection between in silico and in vitro findings.

## 2 Materials and Methods

### 2.1 Materials

In this study, various bioinformatics tools and resources were used to analyze ligand‐protein and protein‐protein interactions and the stability of the compounds. Schrödinger Maestro (version 14.0.134) was utilized, incorporating tools such as Glide for ligand docking, PIPER for protein–protein docking, Prime MM‐GBSA for the calculation of binding free energy, and Desmond for molecular dynamics (MD) simulations. Additionally, ADMETlab was used to assess drug‐likeness and pharmacokinetic properties.

All chemicals and reagents, such as Aβ1‐42 peptides, were acquired from Sigma‐Aldrich Chemical Co. (St. Louis, MO, USA). Fetal bovine serum (FBS), neurobasal medium, antibiotic/antimycotic solution, and Dulbecco's modified Eagle's medium (DMEM) were obtained from Gibco BRL (USA). Plasticware for cell culture was purchased from Nunc, Denmark. We procured fisetin from TCI Chemicals (India) Pvt.

### 2.2 Methods

#### 2.2.1 In Silico Analysis

##### 2.2.1.1 Three‐Dimensional Structure Preparation

The co‐crystallized protein structures of the receptors associated with brain activity, such as autophagy regulators (Beclin1, ULK1, LC3B, Situin1, ATG13, p62/SQSTM1, and p21/CDKN1A), enzymes involved in neurotransmission (AChE), a synaptic density marker (PSD95), the ion channel Na^+^/K^+^‐ATPase, and Ca2^+^‐ATPase, were retrieved from the Protein Data Bank (PDB) (https://www.rcsb.org/). Receptors lacking the cocrystallized structures of those involved in the maintenance of synaptic density, such as synapsin and synaptophysin, were modeled via Alphafold (https://alphafold.com). Moreover, the structure of amyloid beta (Aβ) was retrieved from the PDB for protein–protein docking.

##### 2.2.1.2 Ligand Structure Retrieval and Preparation

The structure of fisetin (PubChem CID: 5281614) was retrieved from the PubChem database (https://pubchem.ncbi.nlm.nih.gov/compound/5281614) in SDF format. These ligand structures were prepared by the LigPrep module, which generates 3D conformations, assigns accurate bond orders, and generates relevant tautomeric states via Epik at physiological pH (7.0 ± 2.0), along with minimizing energy via the OPLS4 force field. This module produced the desired stereoisomers.

#### 2.2.2 Molecular Docking

Molecular docking of fisetin was conducted against brain‐related receptors critical for neurological functions, including targets involved in neurotransmission, autophagy, and synaptic plasticity. Schrödinger Maestro 14.0.134 was used with Glide for ligand docking and PIPER for protein‐protein docking. SiteMap was used to identify potential binding pockets, followed by the generation of a receptor grid. Docking was performed in Glide XP mode to ensure accuracy. Prime MM‐GBSA calculations refined binding affinity predictions by estimating binding free energies with local minimization via the OPLS4 force field and the VSGB 2.1 solvation model. Complexes showing favorable MM‐GBSA scores and interaction profiles were selected for further analysis.

#### 2.2.3 Molecular Dynamics Simulation

The dynamic stability of the selected docked complexes was determined via the method of Desmond in Maestro. The protein‐ligand complex was placed in a water box (TIP3P model) and balanced by the addition of sodium and chloride ions. The system was minimized and equilibrated, and then a 200 ns run was given. The trajectory of the simulation was analyzed on the basis of various parameters, indicating the stability of the complex with respect to time.

#### 2.2.4 Prediction of ADMET Properties

ADMET analysis of fisetin was performed via ADMETlab 3.0 (https://admetlab3.scbdd.com/server/screening). The ligand structure was uploaded to the server in SDF format, where the properties of the drug were predicted. Different physicochemical and key pharmacokinetic properties were estimated along with the evaluation of the compounds' suitability by applying Lipinski's rule of five, which is significant in drug design.

### 2.3 In Vitro Studies

#### 2.3.1 SH‐SY5Y Cell Culture

The human neuroblastoma cell line SH‐SY5Y was obtained from ATCC (USA). Cells were cultured in DMEM/F12 supplemented with 0.2% sodium bicarbonate, 1% antibiotic/antimycotic solution, and 10% FBS. The cultures were maintained in an incubator with 5% CO_2_. The medium was replaced twice a week to ensure nutrient availability and removal of cellular debris. Before experimentation, the cells were terminally differentiated using 10^−5^ M trans‐retinoic acid and 50 ng/mL BDNF in neurobasal medium supplemented with N2 and B27 for 7 days. This differentiation protocol has been employed to inhibit proliferation and induce a mature neuronal phenotype.

#### 2.3.2 Treatment of SH‐SY5Y Cells

SH‐SY5Y cells were divided into the following four treatment groups and treated for 24 h: Group I: SH‐SY5Y control untreated cells, Group II: SH‐SY5Y cells + fisetin, Group III: SH‐SY5Y cells + Aβ1‐42, and Group IV: SH‐SY5Y cells + Aβ1‐42 + fisetin.

A fisetin stock solution was prepared in DMSO at 10 mM and subsequently diluted in DMEM/F12 to obtain working concentrations of 25, 50, 100, 150, and 200 µM. The Aβ1‐42 stock solution was prepared in sterile 0.9% normal saline, followed by aggregation through incubation at 37°C for 4 days at a concentration of 1 mg/mL in PBS [18], and further diluted in medium to obtain working concentrations of 0.5, 1, 1.5, and 2 µM.

### 2.4 Analysis of the Effects of Aβ1‐42 and Fisetin on Cell Viability

SH‐SY5Y cells were cultured in DMEM/F12 medium supplemented with 10% FBS, 0.2% sodium bicarbonate, and 1% antibiotic/antimycotic solution. These cells were then seeded in 96‐well plates at a density of 1 × 10^3^ cells per well and cultured for 24 h. A total of 100 µL of medium containing different concentrations of Aβ1‐42 (0.5, 1, 1.5, and 2 µM), fisetin (25, 50, 100, 150, and 200 µM), and a combination of 1.5 µM Aβ1‐42 and 25 µM fisetin was added to each well. The untreated control cells were also maintained in parallel. After 24 h, the MTT assay was performed to assess cell viability. The purple formazan product produced was dissolved in DMSO, and the absorbance was measured at 570 nm. The reading of the blank was subtracted from those obtained from wells containing cells, and the cell viability was calculated. All the experiments were conducted in triplicate. We determined the cytotoxic doses of Aβ1‐42 and fisetin via this assay.

### 2.5 Activities of Ion Channels

#### 2.5.1 Na^+^/K^+^‐ATPase Activity Assay

The cells were treated with Aβ1‐42 (1.5 µM) and fisetin (25 µM), and these concentrations were selected by a cell viability assay. The protein was isolated from cells using RIPA buffer supplemented with a protease inhibitor cocktail. Na^+^/K^+^‐ATPase activity was assessed with slight modifications to a previously described method. A 1.0 mL reaction mixture consisting of 50 μg of protein and assay buffer (100 mM NaCl, 20 mM KCl, 2 mM ATP disodium salt, and 30 mM Tris‐HCl buffer at pH 7.4) was preincubated at 37°C for 10 min, which allowed ouabain (1.5 mM) to interact with ATPase. After incubation, the reaction was terminated by the addition of 500 μL of 15% (w/v) trichloroacetic acid (TCA). The released inorganic phosphate (Pi) was quantified via the Fiske and Subbarow method [19].

#### 2.5.2 Ca^2+^‐ATPase Activity Assay

The activity of Ca^2+^‐ATPase was measured as previously described [20]. The reaction mixture (1.0 mL) contained 50 μg of protein, 50 mM imidazole‐HCl buffer (pH 7.5), 0.4 mM CaCl_2_, 0.4 mM ATP, and 0.32 mM sucrose. The reaction was stopped by the addition of 0.5 mL of ice‐cold 10% TCA and incubation for 15 min at 37°C. The amount of liberated Pi was analyzed via the Fiske and Subbarow method [19].

### 2.6 Analysis of Oxidative Stress

#### 2.6.1 Pro‐Oxidant Assays

##### 2.6.1.1 ROS Analysis

ROS analysis was performed on the cells from all four experimental groups (Groups I–IV) mentioned above. The levels of ROS, which are indicators of oxidative stress, were assessed using 2′,7′‐dichlorodihydrofluorescein diacetate dye following previously established protocols [21]. Briefly, the cells were washed with DPBS, and dye at a working concentration of 20 µM was added to the cells. The cells were incubated with the dye at 37°C for 30 min in the dark. Following three washes with serum‐free medium, fluorescence was recorded using a multiwell microplate reader at an excitation wavelength of 488 nm and an emission wavelength of 530 nm over 15 min. ROS production in the treatment groups is expressed as a percentage change relative to that in the control group, which was normalized to 100%.

##### 2.6.1.2 Lipid Peroxidation (LPO) Analysis

LPO levels were measured via a thiobarbituric acid reactive substances (TBARS) assay, which was based on the method of Esterbauer and Cheeseman [22] with slight modifications. This assay quantifies malondialdehyde (MDA), a byproduct of LPO, which reacts with thiobarbituric acid (TBA) to form an MDA‐TBA adduct. The resulting pink complex absorbs light at 532 nm. Following protein quantification in each sample, 0.2 mL of cell lysate was mixed with TBA reagent containing 100 mM TBA, 10 mM TCA, and 0.1% Triton X‐100 in distilled water and incubated in a water bath at 95°C for 30 min. After the mixture was allowed to cool, the precipitates were removed by centrifugation at 10,000*g* for 10 min at 4°C. The absorbance of the supernatant was recorded at 532 nm, and the data are expressed as nmol of MDA per mg of protein.

##### 2.6.1.3 Analysis of Protein Carbonyl (PCO) Levels

PCO levels were measured spectrophotometrically in the SH‐SY5Y cells in the entire experimental group after 24 h of treatment with 2,4‐dinitrophenylhydrazine (DNPH). This assay is based on the reaction of carbonyl groups in proteins with DNPH to form stable protein hydrazones, which absorb at 366 nm. The reaction mixture was prepared by mixing 0.2 mL of cell lysate with 3.8 mL of 10 mM DNPH in 2 M HCl [23]. The mixture was incubated in the dark for an hour while shaking. Afterwards, 20% (w/v) TCA was added, and the samples were kept on ice for 10 min and centrifuged at 3500 rpm for 20 min. The pellet was washed three times with ethanol/ethyl acetate solution (1:1) and then dissolved in 1.25 mL of 6 M guanidine‐HCl. The PCO content was determined by measuring the absorbance at 370 nm and using an extinction coefficient of 22,000 M^−1^cm^−1^.

### 2.7 Antioxidant Assays

#### 2.7.1 Superoxide Dismutase (SOD) Assay

The cells were treated with Aβ1‐42 (1.5 µM) and fisetin (25 µM) alone and in combination, and SOD activity was measured via a modified nitro‐blue tetrazolium (NBT) reduction method [24]. The assay mixture consisted of 100 µL working solutions of NBT (2.24 mM) and phenazine methosulfate (1 mM) in phosphate buffer (pH 7.8). To this mixture, 100 µL of the protein sample was added. After incubation at 37°C for 20 min in the dark, the reaction was terminated by adding 100 µL of 50 mM sodium carbonate (Na_2_CO_3_) to each well. The absorbance was recorded at 560 nm via a spectrophotometer. The results are expressed as units of SOD activity per mg of protein.

#### 2.7.2 Catalase (CAT) Assay

Following treatment, CAT activity was determined spectrophotometrically as described previously [25]. The rate of hydrogen peroxide (H_2_O_2_) decomposition was measured by monitoring the decrease in absorbance at 240 nm. The protein concentration of the cell lysates was determined via a bicinchoninic acid assay and adjusted to 1 mg/mL in Tris‐HCl buffer. The reaction mixture, comprising 50 mM Tris‐HCl buffer (pH 7.0), 10 mM H_2_O_2_, and 0.1% sodium azide, was added (100 µL) to each well of a 96‐well plate. Next, 20 µL of diluted lysate was added and mixed by pipetting. The absorbance readings were taken immediately at 240 nm every 30 s for 3 min via a microplate reader. CAT activity was expressed as µmol H_2_O_2_ decomposed per minute per mg protein.

#### 2.7.3 Glutathione (GSH) Assay

The GSH content of the cells treated with Aβ1‐42 (1.5 µM) and fisetin (25 µM) alone and in combination was analyzed as described by Rehman, Kode, and Biswas [26]. GSH reacts with 5,5′‐dithio‐bis(2‐nitrobenzoic acid) (DTNB, Ellman's reagent) to form a yellow‐colored TNB product with a maximum absorbance at 412 nm. Briefly, 200 µL of cell lysate was mixed with 200 µL of 0.15 M KCl and then deproteinized by the addition of 500 µL of metaphosphoric acid. After centrifugation at 3000 rpm for 20 min, 500 µL of the supernatant was combined with 2.0 mL of 0.3 M disodium hydrogen phosphate (Na_2_HPO_4_·2H_2_O) and 500 µL of 10 mM DTNB. The GSH levels are reported as µM per mg of protein.

### 2.8 Gene Expression Analysis

Real‐time quantitative PCR was performed to assess the mRNA expression of autophagy (p62, ULK1, and Beclin1), cell cycle (p21 and p16), neuroprotection, and synapse (Sirtuin1, Synapsin1, PSD95, Synaptophysin, and Keap1) markers. The primer sequences were obtained from OriGene (Table 1) and were standardized by us. Total RNA was isolated from SH‐SY5Y cells from all experimental groups using the TRIzol method. Then, cDNA was synthesized via reverse transcription. qPCR reaction mixtures were prepared with gene‐specific primers along with SYBR Green master mix under optimized amplification conditions: initial denaturation followed by 30 cycles of denaturation, annealing, and extension. The relative expression of the genes was determined via the ΔΔ*C*_t_ method, which was further normalized against the housekeeping gene (β‐actin) and analyzed statistically.

**Table 1:** Primer sequences for qPCR.

S. No	Primer	Forward	Reverse	Catalog no.
1	Beta‐actin	CACCATTGGCAATGAGCGGTTC	AGGTCTTTGCGGATGTCCACGT	HP204660
2	p62	TGTGTAGCGTCTGCGAGGGAAA	AGTGTCCGTGTTTCACCTTCCG	HP207341
3	Synaptophysin	TCGGCTTTGTGAAGGTGCTGCA	TCACTCTCGGTCTTGTTGGCAC	HP206750
4	ULK1	GCAAGGACTCTTCCTGTGACAC	CCACTGCACATCAGGCTGTCTG	HP207064
5	Synapsin‐1	CGATGCCAAATATGACGTGCGTG	AGCATCGCAGAGCCAGTATTGG	HP209791
6	Keap‐1	CAACTTCGCTGAGCAGATTGGC	TGATGAGGGTCACCAGTTGGCA	HP229798
7	Sirtuin‐1	TAGACACGCTGGAACAGGTTGC	CTCCTCGTACAGCTTCACAGTC	HP210213
8	p21	AGGTGGACCTGGAGACTCTCAG	TCCTCTTGGAGAAGATCAGCCG	HP200369
9	Beclin‐1	CTGGACACTCAGCTCAACGTCA	CTCTAGTGCCAGCTCCTTTAGC	HP207225
10	PSD95	TCCACTCTGACAGTGAGACCGA	CGTCACTGTCTCGTAGCTCAGA	HP205263
11	p16	CTCGTGCTGATGCTACTGAGGA	GGTCGGCGCAGTTGGGCTCC	HP226191

### 2.9 Statistical Analysis

All the data are presented as the mean ± standard error of the mean from three independent experiments (*n* = 3). Comparisons between two groups were performed via Student's *t*‐test, whereas comparisons among multiple groups were conducted via one‐way analysis of variance. Statistical analyses were performed via GraphPad Prism 9 (version 9.3.1), and a *p*‐value < 0.05 was considered statistically significant.

## 3 Results

### 3.1 Molecular Docking Analysis

**Figure 1:**
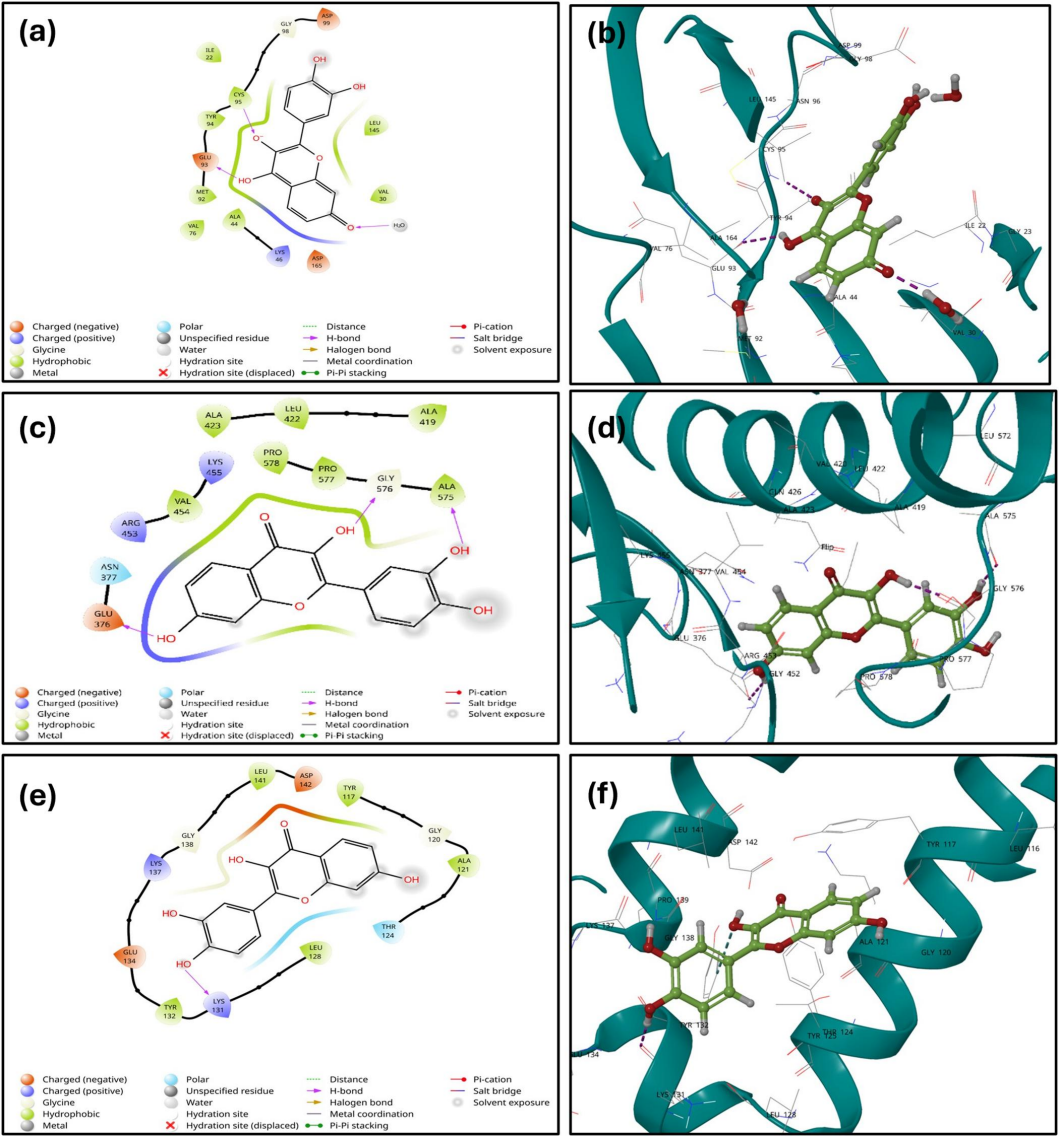
Molecular docking analysis of ULK1, p21, and synaptophysin with fisetin. Two‐dimensional interaction diagrams and three‐dimensional binding poses of fisetin with (a, b) ULK1, (c, d) p21, and (e, f) synaptophysin, indicating the interacting amino acid residues and types of non‐covalent contacts, as shown in the legend (hydrogen bonds, hydrophobic interactions, and other contacts).

**Figure 2:**
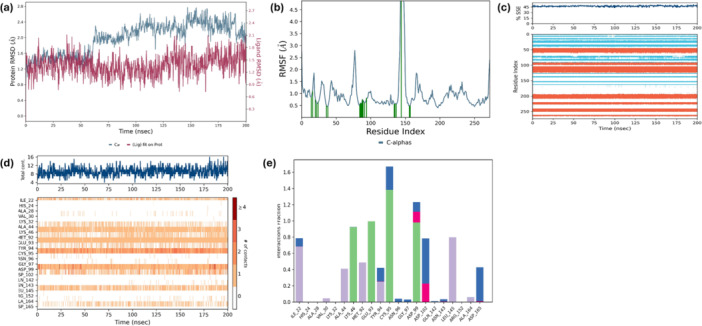
MD simulation analysis of ULK1 in complex with fisetin. (a) Protein–ligand RMSD plot showing the stability of ULK1 and the fisetin ligand over the simulation timeline. (b) Protein RMSF profile illustrating per‐residue fluctuations across the ULK1 sequence. (c) Protein secondary structure element (SSE) timeline depicting the proportion and location of alpha helices, beta strands, and other structural features over the course of the simulation. (d) Timeline of protein–ligand contacts, indicating the number and persistence of interactions between ULK1 residues and fisetin. (e) Histogram summarizing the frequency and types of contact observed between ULK1 residues and fisetin throughout the simulation.

The protein‐protein docking of Aβ yielded favorable PIPER scores of −295.64 with ULK1, −253.15 with p21, and −521.80 with synaptophysin, suggesting the formation of stable and possibly functional complexes with Aβ (Supporting Information S1: Figure 1). The other receptors also demonstrated good binding with Aβ, as highlighted in Supporting Information S1: Figure 2. The binding interactions of each complex are summarized in Supporting Information S1: Table 1.

Moreover, fisetin exhibited strong binding affinity and stability for key autophagy markers (ULK1 and p21) and a synaptic marker (synaptophysin). The ULK1 receptor had a docking score of −8.49 kcal/mol and an MM‐GBSA binding energy (ΔG_bind) of −33.26 kcal/mol, indicating the formation of three hydrogen bonds primarily with the GLU93 and CYS95 residues (Figure 1a,b). Moreover, p21 had a docking score of −5.96 kcal/mol and an MM‐GBSA value of −30.69 kcal/mol, indicating a stable interaction involving the GLU376, GLY576, and ALA575 residues in hydrogen bonding (Figure 1c,d). Synaptophysin is also strongly bound to fisetin, with a binding score of −5.89 kcal/mol and an MM‐GBSA energy of −31.59 kcal/mol, including LYS131 as the key residue involved in hydrogen bonding (Figure 1e,f). In addition, the other receptors listed in Table 2 also exhibited good binding affinity; however, they were relatively unstable (Supporting Information S1: Figure 2a–2h).

**Table 2:** Molecular docking results of fisetin against targets involved in the maintenance of brain function.

Compounds	PDB/UniProt ID	Docking score	MMGBSA dG Bind	Type of interactions	Residues information
Ca^2+^‐ATPase	5ZTF	–10.28	–33.68	H‐bond; Pi‐cation	GLU45; ARG236
AChE	6F25	–9.95	–23.81	H‐bond; Pi‐Pi stacking	TYR334; PHE331; ARG289; SER 286
ULK1	4WNO	–8.49	–33.26	H‐bond	GLU93; CYS95
ATG13	5XV4	–8.87	–50.28	H‐bond	ASN52, ILE47
Sirtuin1	5BTR	–7.86	–47.15	H‐bond	LYS444; ASP298
Na^+^/K^+^‐ATPase	8D3W	–7.89	–46.68	H‐bond	THR368; THR607; ARG541; VAL542; SER474
KEAP1	4IFN	–7.34	–43.75	H‐bond; Pi‐Pi stacking	TYR334; SER508; TYR572
p62	1PFJ	–4.46	–26.88	H‐bond; Pi‐Pi stacking; Pi‐cation	LYS18; HIE78; ASP21
p21	2JOI	–5.96	–30.69	H‐bond	GLU376; GLY576; ALA575
Beclin1	2PIL	–5.57	–36.41	H‐bond; Pi‐Pi stacking; Pi‐cation	LYS87; GLN88; ARG91; TRP24; SER25; ALA84
LC3B	5XAC	–5.12	–37.54	H‐bond; Pi‐cation	GLN72; ARG68; LEU73
Synaptophysin	P07825	–5.89	–31.59	H‐bond	LYS131
Synapsin1	P09951	–4.44	–26.01	H‐bond; Pi‐cation	LYS279; ARG238

### 3.2 Molecular Dynamics Simulation

Molecular dynamics simulations of the top‐ranked molecules from the docking studies were performed to evaluate the stability and binding interactions of the ligand with their respective protein targets. Despite favorable docking and MM‐GBSA scores, along with a significantly negative PIPER score, Ca^2+^‐ATPase did not show substantial stability, whereas ULK1, p21, and synaptophysin were found to have strong and stable interactions with fisetin.

Molecular dynamics simulation of the ULK1‐fisetin complex revealed stable protein‐ligand interactions throughout the 200 ns trajectory. The RMSD profiles for both ULK1 and fisetin remained consistent, indicating minimal structural deviations during the simulation. RMSF analysis revealed regions of increased flexibility within the ULK1 sequence, whereas most of the residues exhibited limited fluctuations, suggesting sustained structural integrity. The protein SSE timeline shows the conservation of major secondary structure elements during simulation. Analysis of protein‐ligand contacts revealed that several ULK1 residues, such as ASP99 and TYR93, formed persistent interactions with fisetin, as observed in both the contact timeline and histogram plots. These findings suggest that fisetin binds stably to ULK1, with the aforementioned key residues possibly contributing to the regulatory activity of the ligand (Figure 2a−e).

**Figure 3:**
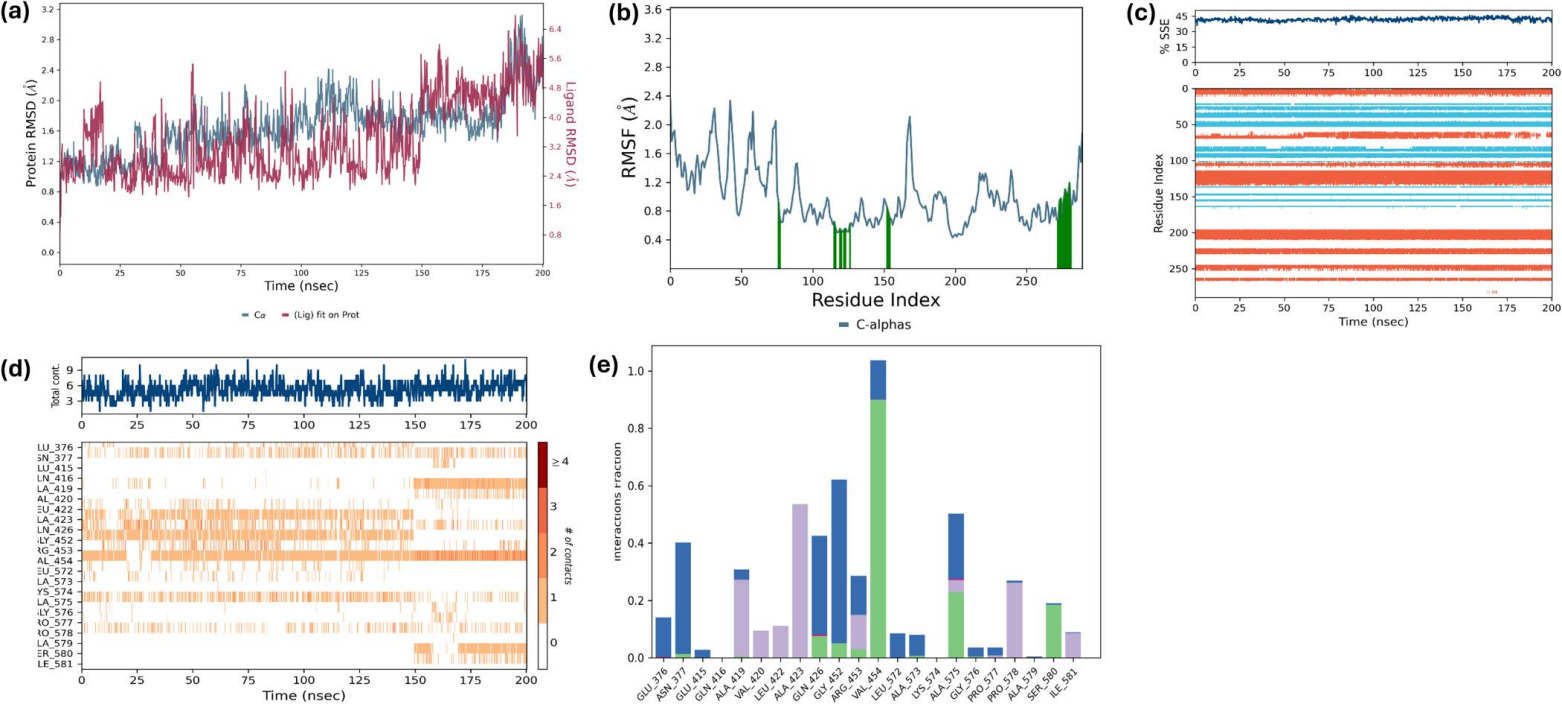
MD simulation analysis of p21 in complex with fisetin. (a) Protein–ligand RMSD plot demonstrating the structural stability of p21 and the bound fisetin molecule across the simulation. (b) Protein RMSF graph depicting residue‐level fluctuations within p21 during the simulation. (c) Protein secondary structure element (SSE) timeline showing the distribution and persistence of structural motifs over time. (d) Protein–ligand contact timeline indicating specific p21 residues and their interaction frequency with fisetin. (e) Histogram of protein–ligand contacts summarizing the main p21 residues involved in fisetin binding events throughout the simulation.

Similarly, the p21‐fisetin complex often shows stability in simulation studies. The protein RMSD gradually increased without exceeding 2.6 Å throughout the course of the simulation, whereas the ligand RMSD was very low, mostly below 2.0 Å during the entire period of 200 ns, which indicates the stability of the ligand binding. According to the RMSF plots, many residues exhibited low flexibility, with the only noticeable deviation observed within some peripheral loops. During the simulation, the secondary structure of p21 was maintained. Analysis of the contact time and histogram data revealed that LYS, ALA, GLY, PHE, and LEU were involved in establishing frequent interactions with fisetin (Figure 3a−e).

**Figure 4:**
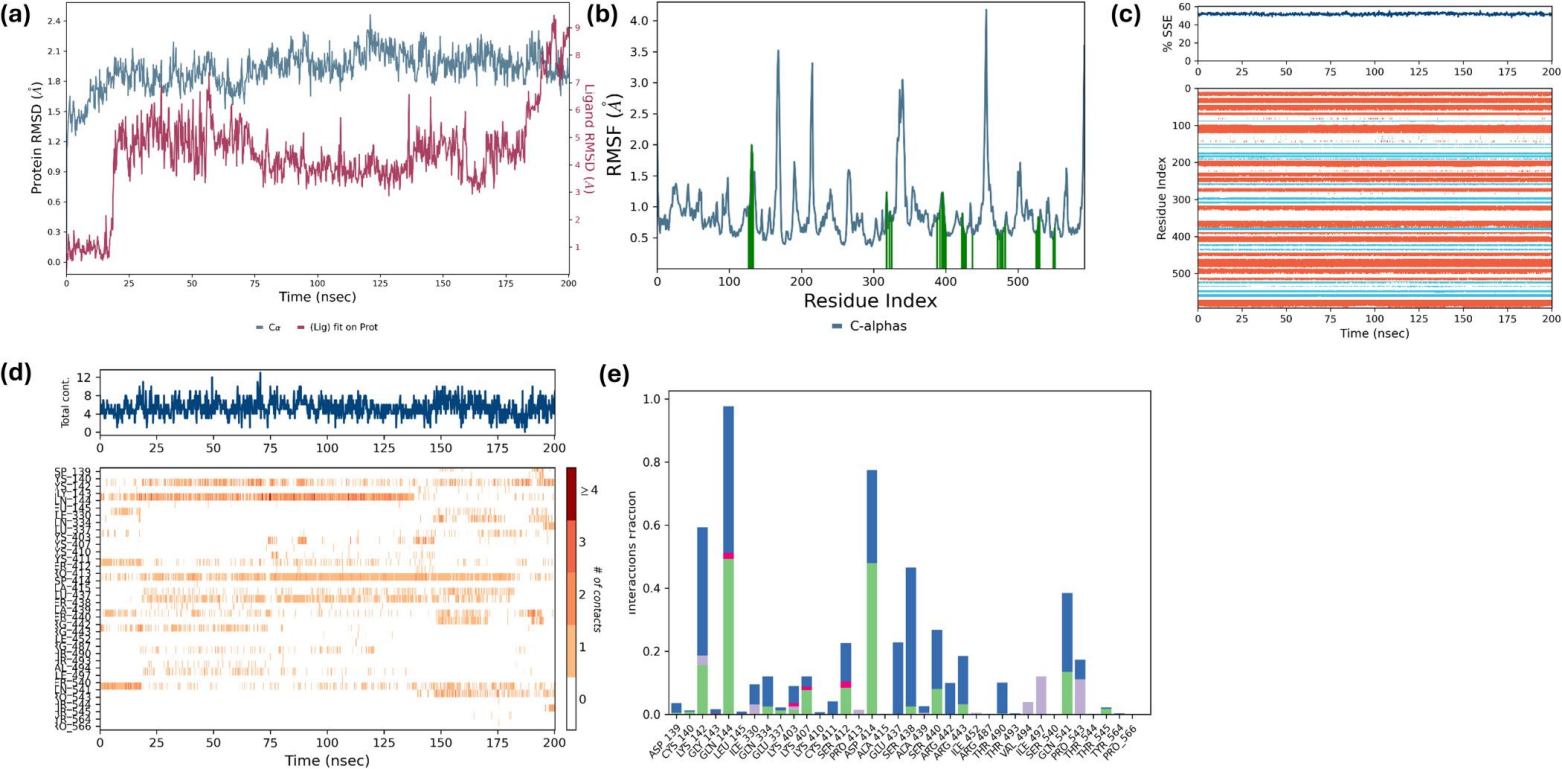
MD simulation analysis of synaptophysin in complex with fisetin. (a) Protein–ligand RMSD traces showing the stability of synaptophysin and the fisetin ligand throughout the simulation. (b) Protein RMSF plot capturing residue‐wise fluctuations in synaptophysin expression during the trajectory. (c) Protein secondary structure element (SSE) timeline, displaying the evolution and persistence of α‐helices, β‐strands, and other motifs across the simulation time. (d) Protein–ligand contact timeline, depicting key synaptophysin residues and the frequency of their interactions with fisetin. (e) Histogram summarizing the principal synaptophysin residues involved in fisetin binding, visualized by interaction frequency over the course of the simulation.

In the synaptophysin‐fisetin complex, the protein RMSD was stable after initial equilibration, and the ligand RMSD remained low for most of the simulations. RMSF analysis revealed that binding site residues remained particularly stable, whereas select loops were more flexible. The protein retained its secondary structure with no significant loss of α‐helix or β‐sheet elements during the MD run (Figure 4a−e).

Taken together, the MD results for all three systems demonstrated that fisetin forms persistent, stable complexes with each protein target, with both the global protein structure and local ligand binding interactions maintained over long simulation timescales.

### 3.3 Drug Profiling

ADMET analysis revealed that the compound exhibited poor intestinal absorption as indicated by low Caco‐2 (−5.759) and MDCK (−4.871) permeability, although the PAMPA results suggested moderate passive diffusion. Distribution studies indicated high plasma protein binding 95.3%, a low distribution volume of −0.743, and poor blood‐brain barrier penetration, indicating that the compound remains largely confined to plasma. High metabolic stability was detected in human liver microsomes; however, the compound strongly inhibited CYP1A2, CYP3A4, and CYP2C8, suggesting a major risk of drug–drug interactions despite not being a substrate of these enzymes. Analysis of excretion predicted a moderate clearance of 11.159 mL/min/kg with a relatively short half‐life of 1.732 h. Toxicity predictions revealed minimal hERG inhibition (0.079), a low probability of drug‐induced neurotoxicity (0.025), and moderate aquatic toxicity (LC50DM = 4.537; LC50FM = 4.178). Overall, the compound demonstrated favorable metabolic stability and low intrinsic toxicity but limited absorption and potential drug‐drug interaction liabilities.

Additionally, fisetin has a molecular weight of 286.05, with 4 hydrogen bond donors (nHDs) and 6 hydrogen bond acceptors (nHAs), and a LogP (octanol–water partition coefficient) of 1.621 (Table 3). These values satisfy the criteria for drug likeness as defined by the Lipinski rule of five [27], indicating that fisetin possesses favorable physicochemical properties for oral bioavailability and is likely a suitable drug candidate.

**Table 3:** ADMET analysis of fisetin.

Absorption	Caco‐2 permeability	−5.759
	MDCK permeability	−4.871
	PAMPA	+
Distribution	PPB	95.3%
	VDss	−0.743
	BBB	—
Metabolism	CYP1A2 inhibitor	+++
	CYP1A2 substrate	—
	CYP2C19 inhibitor	—
	CYP3A4 inhibitor	+++
	CYP3A4 substrate	—
	CYP2C8 inhibitor	+++
	HLM stability	+++
Excretion	CL_plasma_	11.159
	T1/2	1.732
Toxicity	hERG blockers	0.079
	Drug‐induced neurotoxicity	0.025
	LC50DM	4.537
	LC50FM	4.178

### 3.4 Effects of Aβ1‐42 and Fisetin on Cell Viability

**Figure 5:**
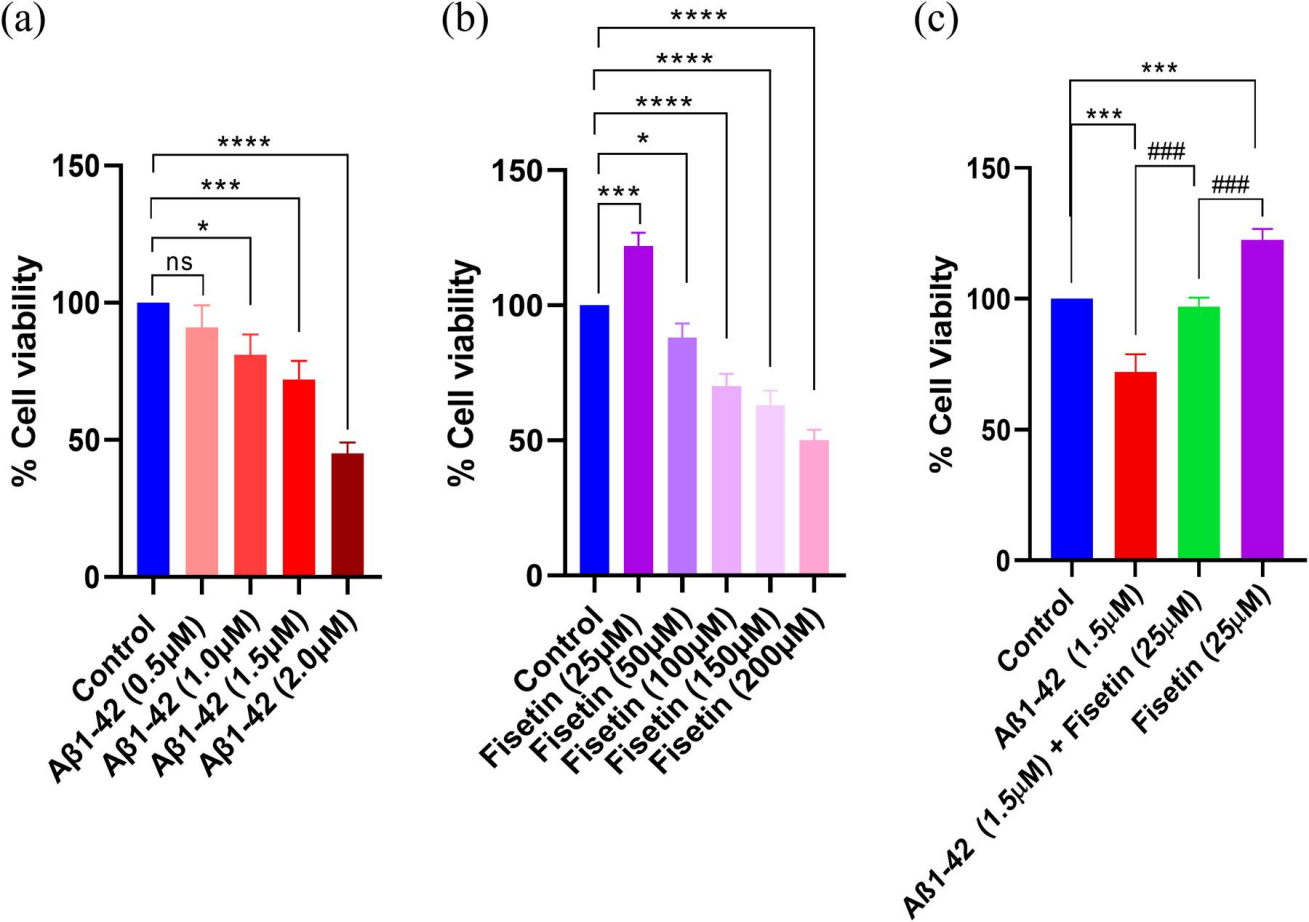
Effects of (a) Aβ1–42, (b) fisetin, and (c) their combination on cell viability. Statistical significance is indicated as *p < 0.05, ***p < 0.001, and ****p < 0.0001 compared with the untreated control group; ###*p* < 0.001, between treatment groups.

A dose‐dependent reduction in cell viability was observed upon exposure to Aβ1‐42 compared with the control (set at 100%). The viability decreased with increasing concentrations of Aβ1‐42 at 0.5 µM (91% ± 8%), 1.0 µM (81% ± 7.4%), 1.5 µM (72% ± 6.8%), and 2.0 µM (45% ± 4%), indicating substantial toxicity at higher concentrations (Figure 5a). The data confirm that Aβ1‐42 alone compromises neuronal survival in a dose‐dependent manner. Treatment with fisetin alone led to a dose‐dependent decrease in cell viability except at a lower dose (25 µM). Fisetin was found to show the highest cell viability of 122% ± 4.94% at the low concentration of 25 µM, suggesting that this dose is optimal for further experiments. Moreover, the cell viability decreased significantly to 88% ± 5.33%, 70% ± 4.66%, 63% ± 5.44%, and 50% ± 3.91% after treatment with fisetin at 50, 100, 150, and 200 µM concentrations, respectively, in comparison to the untreated control (Figure 5b). Compared with treatment with Aβ1–42 alone, co‐treatment with fisetin alone significantly restored cell viability, indicating that fisetin preserves cell viability against Aβ‐induced toxicity. These results indicate that lower concentrations of fisetin increase neuronal viability, whereas higher concentrations partially reduce this protective effect. These data confirmed that Aβ1‐42‐mediated dose‐dependent cytotoxicity occurred (Figure 5c).

### 3.5 Fisetin Maintains Na^+^/K^+^‐ATPase Activity

**Figure 6:**
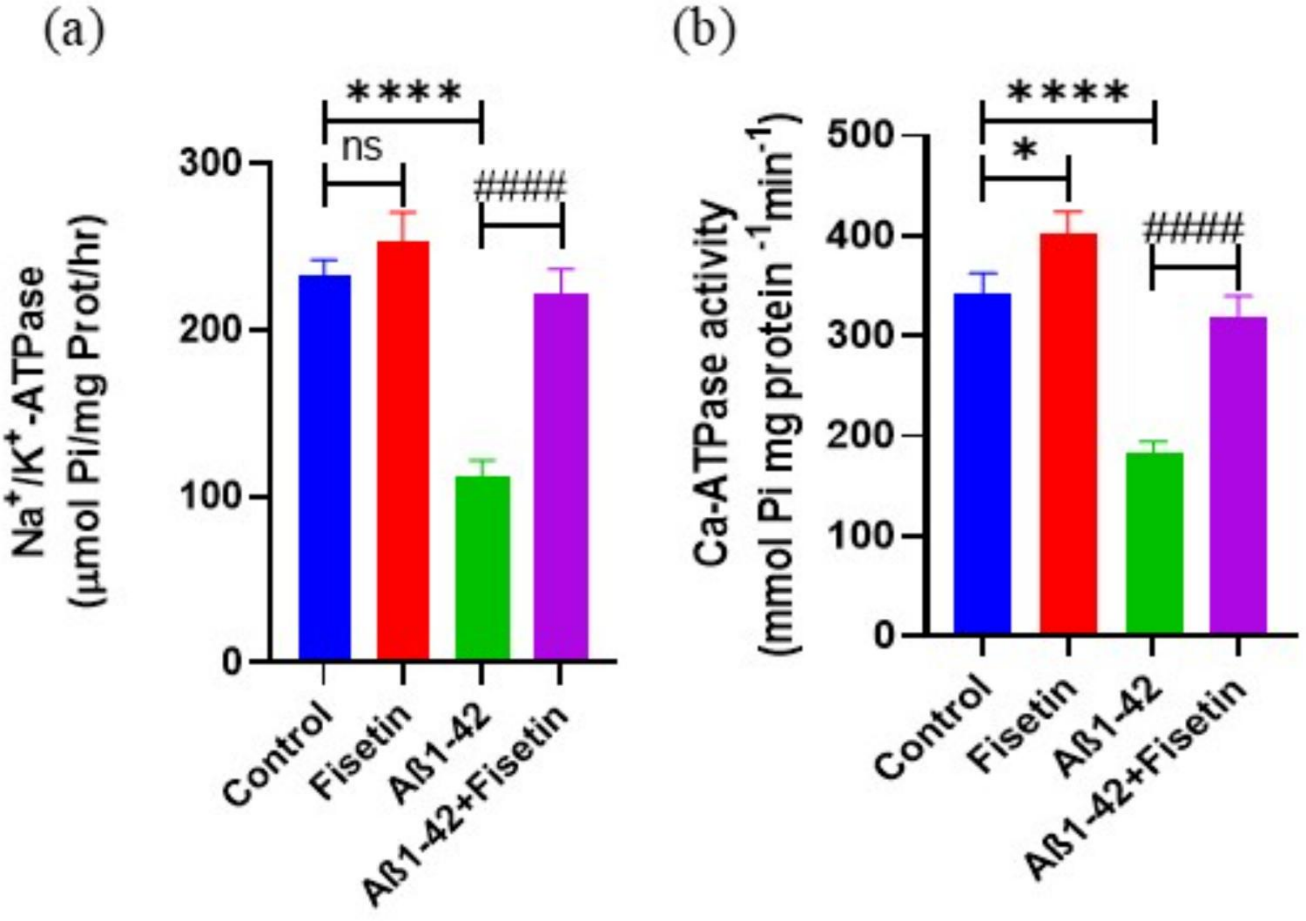
(a) Na^+^/K^+^‐ATPase activity (µmol Pi/mg protein/h) and (b) Ca^2+^‐ATPase activity (nmol Pi/mg protein/min) in control, Aβ1–42‐treated, and fisetin cotreated groups (*p < 0.05, ****p < 0.0001 vs control; and ####*p* < 0.0001 vs. Aβ1–42; ns, not significant).

The Na^+^/K^+^‐ATPase activity in SH‐SY5Y cells was 232 ± 10.2 μmol Pi/mg protein/h in the control group and remained comparable in the fisetin group (253 ± 17.7 μmol Pi/mg protein/h). In contrast, exposure to Aβ1‐42 markedly reduced Na^+^/K^+^‐ATPase activity to 112 ± 9.8 μmol Pi/mg protein/h. The co‐treatment with fisetin significantly attenuated this decrease, resulting in an activity level of 221 ± 15.4 μmol Pi/mg protein/h, which was comparable to the control values. These findings indicate that fisetin effectively counteracts the Aβ1‐42‐induced inhibition of Na^+^/K^+^‐ATPase activity and helps preserve Na^+^/K^+^ ion transport across the plasma membrane (Figure 6a).

### 3.6 Ca^2+^‐ATPase Activity

Ca^2+^‐ATPase activity was 343 ± 19.8 nmol Pi/mg protein/min in control cells and increased to 402 ± 22.2 nmol Pi/mg protein/min following fisetin treatment. Aβ1‐42 exposure significantly decreased Ca^2+^‐ATPase activity to 183 ± 11.4 nmol Pi/mg protein/min, indicating impaired Ca^2+^ extrusion. In the Aβ1‐42+ fisetin group, Ca^2+^‐ATPase activity was restored to 320 ± 19.6 nmol Pi/mg protein/min, indicating marked recovery toward control levels. Together, these data show that fisetin protects Ca^2+^‐ATPase against Aβ1‐42 toxicity, thereby contributing to the maintenance of ionic homeostasis (Figure 6b).

### 3.7 Alleviation of Pro‐Oxidant Parameters by Fisetin

**Figure 7:**
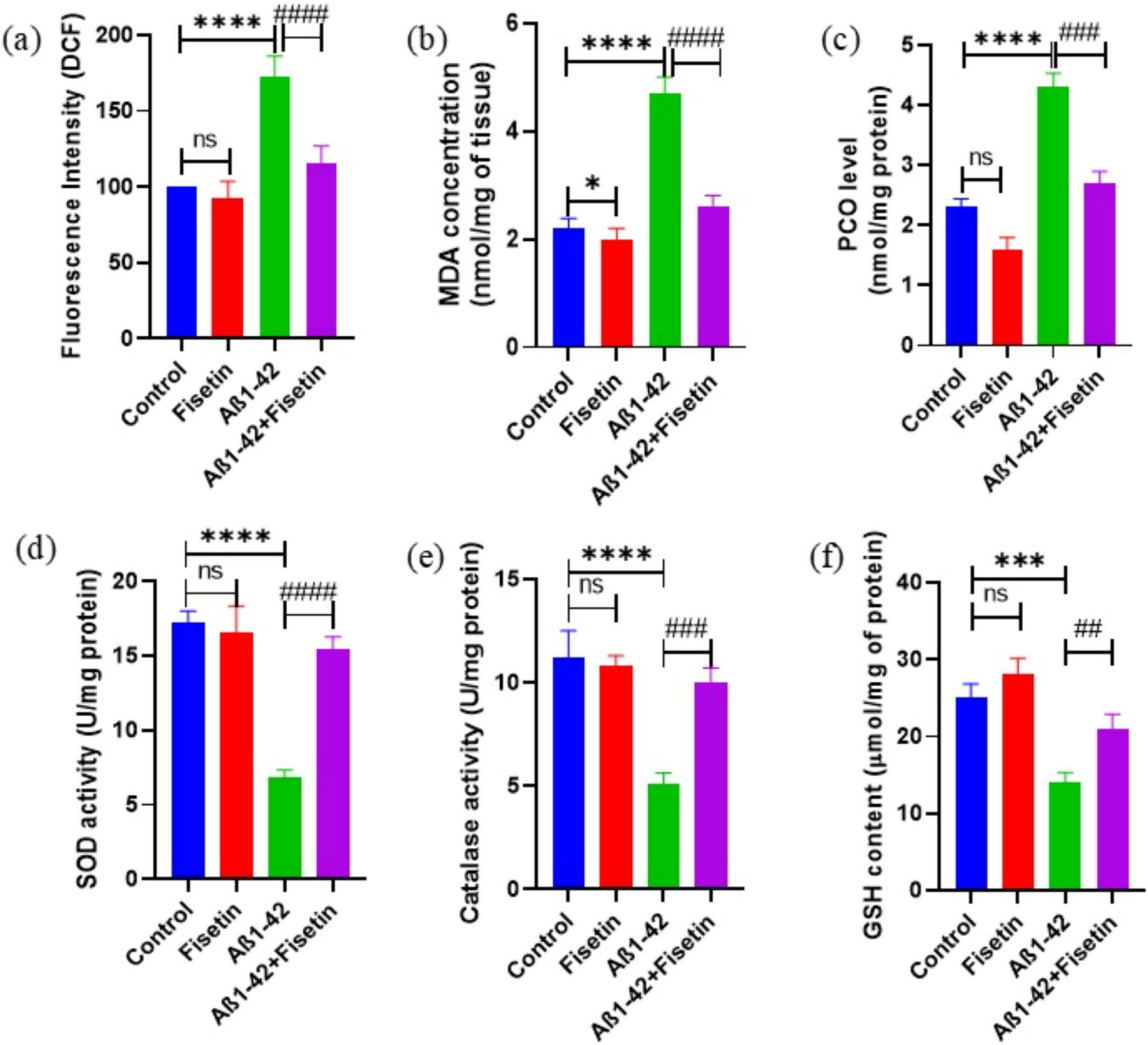
Effect of fisetin on Aβ1–42‐induced oxidative stress markers. (a) Intracellular ROS levels (DCF fluorescence), (b) lipid peroxidation (MDA), (c) protein oxidation (PCO), (d) SOD activity (U/mg protein), (e) catalase activity (U/mg protein), and (f) GSH levels (µmol/mg protein) in the control, Aβ1–42‐treated, and fisetin cotreated groups. The data are presented as the mean ± SEM. Statistical analysis was performed using one‐way ANOVA (*p < 0.001, ****p < 0.0001 vs control; and ##*p* < 0.01, ###*p* < 0.001, ####*p* < 0.0001 vs. Aβ1–42; ns, not significant).

Compared with that in the control group, the intracellular ROS level in the Aβ1‐42 group (172% ± 14.2% of that in the control group) was higher, indicating pronounced oxidative stress. The co‐treatment with fisetin significantly reduced the percentage of ROS to 116% ± 11.1%, whereas treatment with fisetin alone resulted in a value of 93% ± 10.7%, which was slightly lower than that of the control. Similarly, LPO was increased by Aβ1‐42, with the MDA concentration increasing from 2.2 ± 0.19 in the control group to 4.7 ± 0.31 nmol/mg, whereas the levels in the Aβ1‐42+ fisetin and fisetin groups decreased to 2.6 ± 0.21 and 2.2 ± 0.20 nmol/mg, respectively. The protein carbonyl content also increased from 2.6 ± 0.18 nmol/mg protein in the control group to 5.1 ± 0.33 nmol/mg in the Aβ1–42 group, but decreased to 3.0 ± 0.22 and 2.3 ± 0.15 nmol/mg in the Aβ1‐42+ fisetin and fisetin groups, respectively, further confirming the antioxidant effect of fisetin (Figure 7a−c).

### 3.8 Fisetin Restores Antioxidant Levels in SH‐SY5Y Cells

The exposure of Aβ1‐42 significantly decreased SOD activity (6.8 ± 0.54 U/mg protein) in comparison to the control (17.2 ± 0.8 U/mg protein), whereas the treatment with fisetin significantly reversed the Aβ1‐42‐induced reduction in SOD activity (15.4 ± 1.6 U/mg protein). Similarly, CAT activity decreased (5.1 ± 0.53 μmol H_2_O_2_ decomposed/min/mg protein) in the Aβ1‐42 group compared with that in the control group (11.2 ± 1.3 μmol H_2_O_2_ decomposed/min/mg protein). However, fisetin treatment significantly reversed the Aβ1‐42‐induced reduction in CAT activity (10.0 ± 0.5 μmol H_2_O_2_ decomposed/min/mg protein).

The total GSH content decreased in SH‐SY5Y cells following the treatment with Aβ1‐42 (14 ± 1.3 μmol/mg protein) compared with that in control cells (25 ± 1.8 μmol/mg protein). The treatment with fisetin significantly restored the GSH content (21 ± 1.9 μmol/mg protein). Collectively, these data demonstrate that fisetin reduces the levels of Aβ1‐42‐induced prooxidant markers (ROS, MDA, and PCO) while increasing the levels of SOD, CAT, and GSH, suggesting that fisetin has antioxidant activity (Figure 7d−f).

### 3.9 Gene Expression Analysis of Markers Associated With Autophagy, Senescence, and Synapses

**Figure 8:**
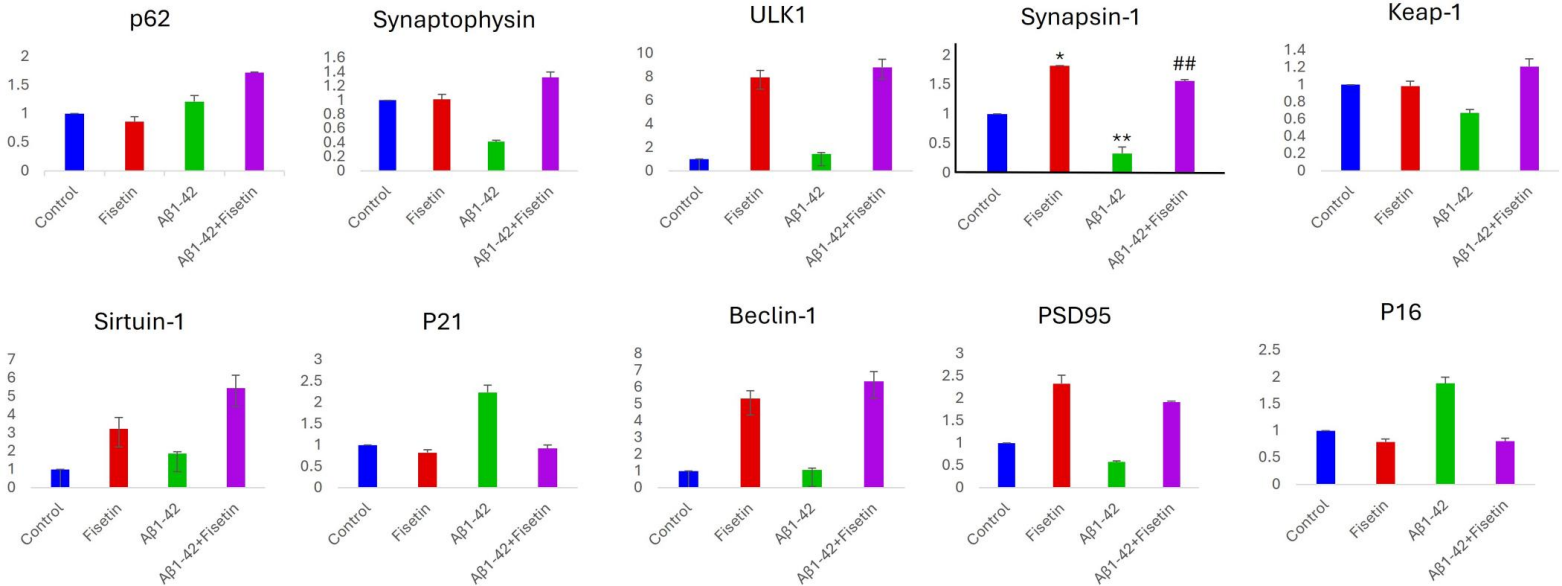
Bar graphs showing the relative mRNA expression levels of p62, ULK1, Beclin1, p21, p16, Sirtuin1, Synapsin1, PSD95, Synaptophysin, and Keap1 in the control, fisetin, Aβ1‐42, and Aβ1‐42+ fisetin groups, as determined by qPCR and normalized to the expression of the housekeeping gene. The data are presented as the means ± SEMs; statistical comparisons were performed via one‐way ANOVA. *p < 0.05, **p < 0.01, ***p < 0.001 versus control; and #*p* < 0.05; ##*p* < 0.01, ###*p* < 0.001 versus Aβ1‐42.

qPCR analysis revealed statistically significant changes in the expression of autophagy, senescence, and synaptic markers across experimental groups. Aβ1‐42 treatment dysregulated these changes, whereas fisetin restored their expression in SH‐SY5Y cells. The expressions of the autophagy markers ULK1 and Beclin1 were upregulated by fisetin (7.92 ± 0.61 and 5.33 ± 0.45 fold, respectively, vs. the control). Moreover, the expression of ULK1 and Beclin1 remained slightly changed in the Aβ1‐42‐treated group (1.44 ± 0.10 and 1.07 ± 0.09 fold, respectively) and was significantly upregulated in the Aβ1‐42 + fisetin group (8.7 ± 0.71 and 6.34 ± 0.59 fold, respectively). In addition, compared with that in the control group, the expression of p62 in the group treated with fisetin (1.21 ± 0.11 fold) increased moderately, whereas that in the group treated with fisetin (1.72 ± 0.01 fold) increased significantly. The expression of the senescence regulators p21 and p16 was induced by Aβ1‐42 (2.23 ± 0.17 and 1.88 ± 0.12 fold, respectively), but fisetin co‐treatment reduced the expression of these genes toward or below the control (0.92 ± 0.08 and 0.803 ± 0.06 fold, respectively), while the expression of Sirtuin1 and Keap1, whose expression was altered by Aβ1‐42 (1.87 ± 0.11 and 0.67 ± 0.04 fold, respectively), was significantly increased by fisetin alone (3.22 ± 0.26 and 0.983 ± 0.059, respectively) and further increased in the Aβ1‐42+ fisetin group (5.43 ± 0.42 and 1.21 ± 0.09 fold, respectively). Consistent with synaptic protection, Aβ1‐42 downregulated the expression of synapsin1, PSD95, and synaptophysin (0.33 ± 0.023, 0.574 ± 0.03, and 0.412 ± 0.02 fold, respectively), whereas fisetin alone upregulated their expression (1.82 ± 0.11, 2.33 ± 0.19, and 1.01 ± 0.07 fold, respectively) and co‐treatment with fisetin (in Aβ1‐42+ fisetin group) restored or exceeded control levels (1.56 ± 0.087, 1.92 ± 0.02, and 1.32 ± 0.08 fold, respectively), indicating that fisetin promotes autophagy, attenuates senescence signaling, and preserves synaptic marker expression in the context of Aβ1‐42 induced alterations. Compared with those in the control group, the levels of ULK1, Beclin1, and synaptic markers in the fisetin alone group slightly increased but remained lower than those in the combination group, whereas the levels of p21 and p16 were comparable to or slightly lower than the Aβ1‐42+ fisetin levels (Figure 8).

## 4 Discussion

In this study, we investigated the neuroprotective potential of fisetin by assessing its effects on ion channel activity, oxidative stress, synaptic plasticity, autophagy, and neurotoxicity in Aβ1‐42‐intoxicated SH‐SY5Y human neuroblastoma cells. The results demonstrated that fisetin enhanced cell viability and provided neuroprotection against Aβ1‐42‐induced toxicity when combined with the modulation of autophagy regulators and synaptic proteins, thereby restoring autophagy, reducing senescence, and preserving synaptic integrity in neuronal cells. However, a concentration‐dependent reduction in cell viability was observed at higher concentrations of fisetin, indicating the need for further toxicity evaluation. In support of this, in silico analysis revealed the stable binding of fisetin to ULK1, p21, and synaptophysin. Additionally, in SH‐SY5Y cells, fisetin enhanced antioxidant defenses, maintained Na^+^/K^+^‐ATPase and Ca^2+^‐ATPase activity, and increased the expression of autophagy and synaptic markers following Aβ1‐42 exposure.

ULK1 initiates autophagy by forming a complex with ATG proteins, followed by autophagosome formation, which leads to cellular clearance of damaged proteins and organelles. Its activity is tightly regulated by nutrient and energy sensors, mainly AMPK and mTORC1. Under conditions of stress or nutrient deprivation, AMPK activates ULK1 through phosphorylation, promoting autophagy. However, mTORC1 inhibits ULK1 under nutrient‐rich conditions. In AD, disrupted AMPK‐ULK1 signaling results in decreased ULK1 activation, leading to impaired autophagy initiation. Consequently, the accumulation of toxic proteins, such as Aβ, damages mitochondria and exacerbates neurodegeneration. Thus, impaired ULK1 function is one of the contributing factors to AD pathology, and enhancing its activity represents a promising therapeutic strategy to restore autophagic flux and neuronal health [28] [29]. Our study demonstrated strong binding of ULK1 with fisetin and Aβ, suggesting that fisetin is a potent therapeutic candidate for AD that targets Aβ‐induced neurotoxicity. These findings were further supported by the results of *in vitro* experiments in SH‐SY5Y cells, which produced consistent results, confirming the neuroprotective effects of fisetin through the modulation of ULK1‐related pathways.

Other proteins, including p21 and synaptophysin, also have strong binding affinities with fisetin and Aβ, indicating their potential roles in modulating neurodegeneration. p21 is a cyclin‐dependent kinase inhibitor that is involved in the regulation of autophagy and AD. Under physiological conditions, it negatively regulates basal autophagy [30]. Mechanistically, inhibition of p21 activates Akt kinase, leading to an increase in intracellular ROS levels due to phosphorylation‐dependent suppression of transcription factor FoxO and a reduction in the expression of ROS scavengers. The resulting accumulated ROS act as a signal to induce autophagy. This p21‐mediated autophagy regulation is independent of p53. Thus, p21 acts as a physiological inhibitor of autophagy by modulating Akt‐ROS signaling, and its suppression enhances autophagic flux. These findings highlight the relevance of this pathway, not only in cancer progression but also potentially in neurodegenerative diseases, where autophagy modulation is key [30]. In AD, upregulated p21 is a common marker of cellular senescence, and its expression is linked to neuronal aging and dysfunction. Moreover, p21 elevation contributes to neurodegenerative pathology by promoting senescent phenotypes in neuronal and glial cells. It further impairs the autophagic clearance of toxic protein aggregates such as Aβ1‐42, exacerbating neuronal loss and cognitive decline. Therefore, the dysregulation of p21 in AD impairs the balance between cellular repair and degradation, making it a key factor at the intersection of senescence, autophagy deficiency, and neurodegeneration [29].

Synaptophysin is a presynaptic vesicle marker, the loss or dysregulation of which indicates both the onset and progression of synaptic failure in AD patients. In the human post‐mortem cortex and hippocampus, the levels of synaptophysin are consistently reduced along with those of other presynaptic proteins, indicating early degeneration of presynaptic terminals that is correlated with cognitive impairment. With the progression of AD, quantitative analyses of synaptophysin in brain sections and synaptosomal fractions demonstrate progressive synapse loss and structural remodeling of residual synapses, thereby linking decreased synaptophysin expression to cognitive decline [31].

ADMET analysis of the compound revealed its pharmacokinetic and safety profile, which is critical for assessing its suitability as a drug candidate. The compound demonstrated high metabolic stability and low predicted toxicity, which are encouraging features for further development. However, its poor intestinal permeability and limited distribution present challenges for oral administration and tissue bioavailability [32]. The strong inhibitory effects on CYP1A2, CYP3A4, and CYP2C8 are of particular concern, as these enzymes play a central role in xenobiotic metabolism, and their inhibition could lead to significant drug–drug interactions if they are co‐administered with commonly prescribed medications [33]. Although the predicted clearance was moderate, the short half‐life points to rapid systemic elimination, which may necessitate structural optimization or formulation strategies to prolong drug exposure. From a safety perspective, the low risk of hERG channel inhibition and neurotoxicity is favorable [34, 35]. Collectively, these findings indicated that the compound exhibits desirable metabolic stability and safety margins; further optimization is required to overcome limitations in absorption, distribution, and CYP‐mediated interactions. These insights not only guide the refinement of this compound but also contribute to the rational design of next‐generation analogs with improved pharmacokinetic and safety profiles.

Na^+^/K^+^‐ATPase, a ubiquitous membrane transporter, maintains cellular homeostasis. In the brain, oxidative metabolism is high; thus, most ATP is consumed to sustain the Na^+^/K^+^ gradients critical for nerve impulse propagation, neurotransmitter release, and ion homeostasis [36]. In AD, Aβ oligomers bind to the neuron‐specific Na^+^/K^+^‐ATPase α3, inhibit its pump activity, trigger N‐type Ca^2+^ channel activation, and induce mitochondrial Ca^2+^ dyshomeostasis, tau abnormalities, and neuronal death. Aβ1‐42 also forms a complex with Na^+^/K^+^‐ATPase α1, disrupting Na^+^/K^+^‐ATPase function and activating Src‐kinase signaling, whereas astrocytic Na^+^/K^+^‐ATPase α2 promotes tauopathy and neuroinflammation; thus, the modulation of Na^+^/K^+^‐ATPase can aid in the mitigation of AD progression [37].

The results of this study indicate that plasma membrane Ca^2+^‐ATPase 2 (PMCA2) in microglia is a key regulator of calcium signaling that modulates Alzheimer‐‘s‐like pathology. Enhanced Ca^2+^ efflux was shown to attenuate microglial Ca^2+^ signaling, shift microglia toward a less inflammatory phenotype, and reduce the amyloid plaque burden in the AD model. Collectively, these findings directly implicate Ca^2+^‐ATPase activity in AD progression, demonstrating that restoring PMCA2‐dependent calcium homeostasis in microglia can ameliorate both neuroinflammation and amyloid pathology [37]. We found that fisetin effectively reversed the Aβ1‐42‐induced suppression of both Na^+^/K^+^‐ATPase activity and Ca^2+^‐ATPase activity, thereby supporting ion channel function and cellular homeostasis. Several studies have shown that fisetin has strong antioxidant effects that protect cells from oxidative stress. High levels of ROS in brain tissue reduce ATP levels and mitochondrial membrane potential, causing DNA damage, LPO, apoptosis, and inflammation, all of which contribute to neurodegenerative diseases. As a polyphenol found in common foods, fisetin reduces oxidative stress, ROS, neurotoxicity, and neuroinflammation while supporting mitochondrial function and inhibiting nitric oxide production [13]. It regulates molecular pathways, such as the Nrf2, PI3K/Akt, protein kinase C, NF‐κB, and MAPK pathways to prevent oxidative damage, inflammation, and cell death, thus protecting neural cells from degeneration and potentially preventing neurodegenerative disorders [38]. Although these studies have been performed, they have not been linked to AD. Our study demonstrated the potential of fisetin in reducing oxidative stress in Aβ1‐42‐treated cells by reducing prooxidant levels and increasing antioxidant levels.

GSH is a crucial antioxidant that neutralizes ROS [39]. Peroxynitrite, a highly reactive oxidant formed by the interaction of excess superoxide anion radicals with NO, contributes to tissue damage [40]. In this study, fisetin significantly increased GSH levels [41] while reducing ROS, NO, and MDA levels in SH‐SY5Y cells. As a potent antioxidant phenylpropanoid, fisetin also protects the liver from the toxic effects of various drugs, including trimethylamine‐N‐oxide, thioacetamide, carbon tetrachloride, methotrexate, and N‐acetyl‐p‐aminophenol [42].

Sirtuin1, a promising blood biomarker for the early diagnosis of AD, is significantly reduced in AD patients. Lower serum Sirtuin1 levels are associated with cognitive decline and greater disease severity. Sirtuin1 exerts neuroprotective effects by regulating Aβ and tau pathologies. Fisetin has been shown to activate Sirtuin1, thereby enhancing its protective functions by facilitating Aβ clearance, decreasing tau accumulation, and reducing oxidative stress and inflammation. These findings indicate that fisetin helps restore Sirtuin1 activity in AD patients. Consequently, fisetin‐mediated modulation of Sirtuin1 expression underscores its potential as a valuable neuroprotective agent for treating AD [43]. These results are similar to our findings, in which fisetin restored the Sirtuin1 level. Fisetin often exerts neuroprotective effects by increasing the expression of synaptic proteins such as PSD95, which enhances synaptic function and memory during neurodegeneration. It promotes autophagy by inhibiting mTOR activity and activating ULK1, thereby facilitating autophagic flux through the regulation of key components such as the autophagy receptor p62.

Furthermore, fisetin diminishes cellular senescence and inflammation by decreasing the levels of markers such as p21 and p16, helping to reduce neuronal apoptosis and chronic neuroinflammation. These findings are consistent with those of our study, which revealed that fisetin promotes neuronal survival, improves cognitive function, and offers protection against neurodegenerative damage [38]. Fisetin significantly reduces the expression of the senescence‐associated proteins p21 and p16, suggesting that it plays a role in neuroprotection by mitigating senescence‐related pathways [44]. It also modulates the levels of p62, a key autophagy cargo receptor. It activates the transcription factors TFEB and Nrf2, which promote the expression of autophagy and lysosomal genes, including p62. Treatment with fisetin initially increased the protein level of p62 in cortical neurons, indicating enhanced production. However, this is followed by a rapid decrease in p62 levels, reflecting its consumption during active autophagic degradation. Thus, fisetin stimulates autophagy, and the transient increase followed by reduction in p62 protein levels highlights that fisetin induces efficient autophagic flux, facilitating the clearance of phosphorylated tau protein and potentially other substrates through the autophagy‐lysosome pathway. The role of fisetin in autophagic clearance is critical for its neuroprotective effects [14].

KEAP1, a central regulator of the oxidative stress response, is found localized within neuronal and glial cytoplasmic inclusions characteristic of AD, including neurofibrillary tangles. KEAP1 interacts with the autophagy‐related protein p62, with both proteins present in similar brain fractions from AD patients. Despite increased expression of Nrf2 target genes in AD brains, the KEAP1‐p62 interaction appears to contribute to cytoplasmic inclusion formation and plays a significant role in oxidative stress responses during AD pathogenesis, indicating that KEAP1 is involved in the molecular mechanisms underlying neurodegeneration in AD [45].

Synapsin1, a key presynaptic phosphoprotein, is also altered in the case of AD. In silico analysis revealed that synapsin1 is the hub gene involved in the disruption of synaptic function and cognitive processes in AD patients. The results of this study revealed that synapsin1 overexpression improved memory and reduced neuroinflammation in the rat AD model. Synapsin1 activates the cAMP signaling, increases the phosphorylation of CREB and PKA, and promotes neurotransmitter release. When synapsin1 is overexpressed, the expression of oxidative stress markers and inflammatory cytokines significantly decreases. Moreover, bioinformatics and structural analyses revealed key binding pockets and interaction sites of synapsin1 with other synaptic and signaling proteins, suggesting molecular mechanisms underlying its protective effects. This study highlights synapsin1 as a promising target for AD treatment by restoring synaptic function and reducing neuroinflammation. In our study, the expression of synapsin1 increased, suggesting that synapsin1 may ameliorate AD [46].

Beclin1 is a key autophagy protein that initiates autophagosome formation by participating in the class III PI3K complex, which is responsible for the nucleation and elongation of the autophagic membrane. In AD, Beclin1 expression is reduced, leading to impaired autophagy, accumulation of toxic amyloid‐beta and tau aggregates, and neuronal degeneration. Dysfunction of Beclin1‐related autophagy also disrupts retrograde transport and lysosomal function in neurons, exacerbating neurodegenerative pathology. Enhancing Beclin1‐mediated autophagy is thus considered a promising therapeutic strategy for AD [47]. Our study often aligns with these studies, indicating increased expression of Beclin1 in AD‐like conditions upon treatment with fisetin.

These gene expression findings are consistent with those of previous studies, in which fisetin was shown to restore PSD‐95 toward control levels and upregulate SIRT1, supporting synaptic integrity and activating SIRT1‐dependent antioxidant and anti‐inflammatory signaling. It also increased Nrf2, enhanced brain antioxidant capacity, and reduced ROS, LPO, neuroinflammation, and neuronal damage [48, 49]. Moreover, it reduces p16Ink4a expression in multiple tissues and decreases p21 levels, indicating a senolytic effect that preferentially targets p16‐high senescent cells while mitigating p21‐associated senescence [50].

This data indicates that Aβ1‐42 disrupts autophagy, triggers cellular senescence, and reduces the expression of synaptic markers in SH‐SY5Y cells. The treatment with fisetin increased the expression of autophagy‐related genes, decreased the expression of senescence markers, and restored the expression of synaptic genes, highlighting its neuroprotective effects. These results further indicate that fisetin effectively mitigates Aβ‐induced toxicity by activating cellular clearance mechanisms, inhibiting senescence pathways, and maintaining synaptic integrity, supporting its potential as a therapeutic agent for neuroprotection. Collectively, these mechanisms contribute to mitigating Aβ1‐42‐induced neurotoxicity.

## 5 Conclusion

In silico studies revealed the stable binding of fisetin to key neuronal receptors and proteins involved in AD pathology, including ULK1, p21, and synaptophysin. Furthermore, ADMET profiling highlighted favorable pharmacokinetic and safety profiles despite certain limitations in absorption and potential drug interaction risks. This study further demonstrated that fisetin has significant neuroprotective effects against Aβ1‐42‐induced neurotoxicity by preserving ion transporter functions, reducing oxidative stress, and maintaining the expression of the genes related to autophagy, senescence, neural, and synaptic markers.

## Author Contributions

**Charu Jaiswal:** writing – original draft, validation, methodology, data curation. **Ishika Singh:** formal analysis and editing. **Abhishek Kumar Singh:** conceptualization, review and editing, investigation, formal analysis, supervision, resources, funding acquisition.

## Conflicts of Interest

The authors declare no conflicts of interest.

## Supporting information


Supporting File 1

Supporting File 2

## Data Availability

All the data supporting the findings of this study are available within the paper.
