# Supplementary material for: Fisetin Attenuates Amyloid‐Beta‐Induced Neurotoxicity in Human Neuroblastoma SH‐SY5Y Cells: Integrating In Silico Target Prediction and In Vitro Validation
Source: J Biochem Mol Toxicol. 2026 Aug 17;40(9):e71076. doi: 10.1002/jbt.71076 (PMC13480354; doi:10.1002/jbt.71076)
Supplement: Supplementary file 1 [file JBT-40-e71076-s001.docx]

**Figure S1:** Two-dimensional interaction diagrams indicating molecular docking of (a) ULK1, (b) p21 and (c) synaptophysin with Aβ

(a)

(c)

(b)

**Figure S2:** Two-dimensional interaction diagrams indicating molecular docking of (a) Ca^2+^-ATPase, (b) Na^+^/K^+^-ATPase, (c) Synapsin1, (d) Beclin1, (e) ATG13, (f) Sirtuin1, (g) p62, (h) LC3b with Aβ

(d)

(c)

(b)

(a)

(h)

(g)

(f)

(e)

**Figure S3:** Two-dimensional and three-dimensional interaction diagrams of (a) Ca^2+^-ATPase, (b) Na^+^/K^+^-ATPase, (c) Synapsin1, (d) Beclin1, (e) ATG13, (f) Sirtuin1, (g) p62, (h) LC3b with Fisetin

(a-II)

(a-I)

(b-II)

(b-I)

(d-II)

(d-I)

(c-I)

(c-II)

(f-I)

(f-II)

(e-II)

(e-I)

(h-I)

(h-II)

(g-II)

(g-I)
