# Supplementary material for: Fisetin Attenuates Amyloid‐Beta‐Induced Neurotoxicity in Human Neuroblastoma SH‐SY5Y Cells: Integrating In Silico Target Prediction and In Vitro Validation
Source: J Biochem Mol Toxicol. 2026 Aug 17;40(9):e71076. doi: 10.1002/jbt.71076 (PMC13480354; doi:10.1002/jbt.71076)
Supplement: Supplementary file 2 [file JBT-40-e71076-s002.docx]

**Table S1:** Molecular docking results of Amyloidβ1-42 using BioLuminate

| **Compounds** | **PIPER Pose score** | **Type of interactions** | **Residues information** |
| --- | --- | --- | --- |
| Ca^2+^-ATPase | -699.61 | Pi-Pi stacking | PHE73; HIS6 |
| NaK_ATPase | -670.52 | Pi-Pi stacking | PHE73; HIS6 |
| KEAP1 | -562.44 | H-bonds | HIS13; PHE182 |
| Synaptophysin | -521.80 | H-bond;  Pi-Pi stacking | TYR278; PHE28; TYR10; PHE20 |
| Synapsin1 | -365.48 | H-bond;  Salt bridges | ARG7; ARG194; ALA42; GLY38 GLU3 |
| Beclin1 | -316.79 | H-bond | ARG100; GLN125; ASN197; PHE20; TYR10; GLY37 |
| ULK1 | -295.64 | H-bond | ALA179; HIS6 |
| Sirtuin1 | -264.59 | H-bond | ASN145; HIE170; HIE14; VAL39; ILE41 |
| p21 | -253.15 | H-bond | ARG371; GLN309; HIS6; ARG5 |
| AChE | -244.49 | H-bond;  Salt bridges | LYS530; GLU22 |
| ATG13 | -242.71 | H-bond | ARG139; ASP23 |
| p62 | -157.49 | H-bond;  Salt bridges | ASP94; LYS16; HIS13 |
| LC3B | -147.56 | Pi-Pi stacking | HIS27; PHE52; HIS14; HIS13 |
| KEAP1 |  | H-bond | PHE182; HIS13 |
| PSD95 | -156.87 | H-bond | HIS596; GLN15 |
| Sirtuin1 | -264.59 | H-bond | HIS14; ASN145; HIE170; VAL39; ILE41 |
